# 

**Published:** 1845-11

**Authors:** 


### CONTENTS

### OF NO. V.

---

### ORIGINAL COMMUNICATIONS.

### ESSAYS AND CASES.

Art. I.—Thoughts on the Difficulty of acquiring accurate knowledge in Practical Medicine: with special reference to the French Schools; being an Extract from the Inaugural Address before the Medical Society of Tennessee. By A. H. Buchanan, M.D., President of the Soctety. - - - - - - - - - - - - - - 369

Art. II.—An Account of Epidemic Metritis. By Francis H. Gordon, M,D., of Wilson county, Tennessee. - - - - - - - 386

Art. III.—Case of Total Blindness, occurring during the admtnistration of large doses of the Sulphate of Quinine. Reported by Henry C. Lewis, of Yazoo City, Mississippi. - - - - - - - 396

Art. IV,—Remarks on a Case of an Acephalous Monster. Read before the Medical Society of Tennessee at its Annual Meeting, on Thursday, 2d May, 1844. By J. E. Manlove, M.D., of Davidson county, Tennessee. - - - - - - - - - - - - 401

Art. V.—Case of Extraordinary Fungous Disease of the Gums, its constitutional effects, and successful treatment. By Henry West, M.D., of St. Clairsville, Ohio. - - - - - - - - - - 405

Art. VI.—A Case of Encysted Placenta, from the Hour-Glass Contraction of the Uterus, the Placenta being unnaturally attached to the Fundus Uteri by a dense cord of cellular substance. By Dr. M. H. Fee, of Leatherwood, Indiana. - - - - - - - - - 408

Art. VII.—Cases Surgical and Obstetrical. By Doctor J. Emery, of Woodville, Missouri. - - - - - - - - - - - - 410

### REVIEWS.

Art. VIII—The Anatomy and Diseases of the Breast. By Sir Astley Cooper, Bart., F.R.S., Sergeant Surgeon to his Majesty; Consulting Surgeon to Guy's Hospital; Lecturer on Anatomy and Surgery, &c., &c., &c· - - - - - - - - - - 412

Art. IX.—The Principles and Practice of Obstetric Medicine and Surgery, in reference to the Process of Parturition. By Francis H. Ramsbotham, M.D., Fellow of the Royal College of Physicians, &c., &c. A new edition from the enlarged and revised London edition. - - - - - - - - - - - - - - - - 420

Art. X.—The First Lines of the Theory and Practice of Surgery, including the Principal Operations. By Samuel Cooper, Senior Surgeon to University College Hospital, and Professor of Surgery in the same College, etc. With Notes and Additions; by WILLARD PARKER, M.D., Professor of Surgery in the College of Physicians and Surgeons in the University of the State of New York, &c. 420

ART. XI.—The Medical Remembrancer, or Book of Emergencies; in which are concisely pointed out the immediate remedies to be adopted in the first moments of danger from Poisoning, Drowning, Apoplexy, Burns, and other accidents: with the Tests for the principal Poisons, and other Useful Information. By EDWARD B. L. SHAW, M.R.C.S. & L.A.S., one of the Surgeons to the Royal Humane Society, and Assissant Apothecary to St. Bartholomew's Hospital. - - - - - - - - - - - - 425

ART. XII.—A Dictionary of Terms used in Medicine and the Collateral Sciences. By RICHARD D. HOBLYM, A. M., Oxon. Revised, with numerous additions, by ISAAC HAYS, M.D., Editor of the American Journal of the Medical Sciences. - - - - - 428

ART. XIII.—The Half-Yearly Abstract of the Medical Sciences. Edited by W. H. RANKING, M.D., Cantab., Physician to the Suffolk General Hospital. - - - - - - - - - - - 428

### SELECTIONS FROM AMERICAN AND FOREIGN JOURNALS

Cases of Poisoning by Belladonna. - - - - - - - - - 429
On Bougies of Slippery Elm Bark. - - - - - - - - 433
Medical Matters, &c., in Geneva. - - - - - - - - - 434
Poisoning by a Small Dose of the Muriate of Morphia applied Externally. 436
Extreme Mercurial Salivation. - - - - - - - - 436
Case of Congenital Malformation of the Heart. - - - - - - 438
On the Decrease of Disease effected by the Progress of Civilization. - 441
Passage of Metallic Mercury into the Blood and other Organs of the body. 448
Gout treated with Belladonna and Naphtha. - - - - - - 449
On the quantity of Carbonic Acid exhaled from the Lungs in 24 hours. 449
Effect of Warm Climate on Pulmonary Diseases. - - - - - 449
Treatment of Infantile Gastric Fever. - - - - - - - - - 450
Remittent Fever. - - - - - - - - - - - - 450
Diabetes—the effect of Cllmate in its treatment. - - - - 450

### ORIGINAL INTELLIGENCE.

National Medical Convention in the City of New York. - - - - 452
Southern and Western Convention at Memphis. - - - - 453
Mississippi Valley Association of Dental Surgeons. - - - - 454
Watson's Lectures on the Practice of Physic. - - - - - - 455
Meteoric Stones. - - - - - - - - - - - - - 455
Heberden's Commentaries. - - - - - - . - - - 457
Improved Life Preserver. - - - - - - - - - - - 458
Harrison's Therapeutics and Materia Medica. - - - - - - 459
Medical Institute of Louisville. - - - - - - . - - 459
Epitome, in English, of Hippocrates and Galen. - . - - 460
Niel on the Arteries. - - - - - - - - - - - - 460

